# Usage Pattern and Nicotine Delivery during Ad Libitum Consumption of Pod E-Cigarettes and Heated Tobacco Products

**DOI:** 10.3390/toxics11050434

**Published:** 2023-05-05

**Authors:** Andrea Rabenstein, Anna Rahofer, Jochen Vukas, Benedikt Rieder, Kristin Störzenhofecker, Yvonne Stoll, Nestor Burgmann, Elke Pieper, Peter Laux, Andreas Luch, Tobias Rüther, Nadja Mallock-Ohnesorg

**Affiliations:** 1 Department of Psychiatry and Psychotherapy, University Hospital, Ludwig Maximilian University of Munich (LMU), Nussbaumstraße 7, 80336 Munich, Germany; anna.rahofer@med.uni-muenchen.de (A.R.); benedikt.rieder@med.uni-muenchen.de (B.R.);; 2 Department of Social Services, Katholische Hochschule Nordrhein-Westfalen, Standort Köln, Wörthstraße 10, 50668 Cologne, Germany; 3 German Federal Institute for Risk Assessment (BfR), Department of Chemical and Product Safety, Max-Dohrn-Straße 8-10, 10589 Berlin, Germany

**Keywords:** pod e-cigarette, JUUL, heated tobacco product, IQOS, nicotine delivery, ad libitum consumption, puff topography

## Abstract

Many different nicotine delivery products, such as e-cigarettes (e-cigs) or heated tobacco products (HTPs), are available on the market. To better understand these products, it is crucial to learn how consumers use them and how much nicotine they deliver. Therefore, a pod e-cig, an HTP, and a conventional cigarette (CC) were each used by 15 experienced users of the respective product category for 90 min without special use instructions (“ad libitum”). Sessions were video recorded to analyze usage patterns and puff topography. At defined time points, blood was sampled to determine nicotine concentrations, and subjective effects were inquired about using questionnaires. During the study period, the CC and HTP groups averaged the same number of consumption units (both 4.2 units). In the pod e-cig group, the highest number of puffs was taken (pod e-cig 71.9; HTP: 52.2; CC: 42.3 puffs) with the most extended mean puff duration (pod e-cig: 2.8 s; HTP: 1.9 s; CC: 1.8 s). Pod e-cigs were predominantly used with single puffs or in short clusters of 2–5 puffs. The maximum plasma nicotine concentration was highest for CCs, followed by HTPs, and then pod e-cigs with 24.0, 17.7, and 8.0 ng/mL, respectively. Craving was reduced by all products. The results suggest that the high nicotine delivery known for tobacco-containing products (CCs and HTPs) may not be needed for non-tobacco-containing products (pod e-cigs) to satisfy cravings in experienced users.

## 1. Introduction

Tobacco consumption has been given the status of a “global epidemic” by the WHO due to its negative health impact; for instance, for the increased risk of developing cancer, cardiovascular diseases, lung diseases, and periodontal diseases [1,2,3,4,5,6].

A review from 2017 has estimated the risk of dying early to be three times higher among smokers compared with nonsmokers [7].

In addition to conventional cigarettes (CCs), numerous other forms of nicotine consumption are now available. A very heterogeneous product category is called ENDS (Electronic Nicotine Delivery Systems) and has gained increasing popularity since its introduction, especially among young adults [8]. ENDS include battery-powered products such as heated tobacco products (HTPs) and e-cigarettes (e-cigs). Both contain nicotine and produce an aerosol that is inhaled. To have a potential benefit for addicted smokers that are unable to quit smoking, ENDS should satisfy craving sufficiently to avoid relapse to CC smoking. Thus, the nicotine delivery of ENDS is an important factor when assessing the risk of these products. A rapid invasion of nicotine into the blood in the acute phase of consumption is discussed to enhance the addictiveness of the product [9,10,11]. If the nicotine delivery is too low, product-use craving might not be satisfied, potentially leading to an increased use of the product [12]. Such compensation could increase the exposure to hazardous substances [13].

The product group of e-cigs can be divided into product generations with different features, but their general structure is mostly common. They consist of a battery, a heating coil with wick, a tank for the liquid, the liquid to be vaporized, and a mouthpiece [14]. At first, this product category started with disposable, low-power products with low nicotine delivery—so-called “cigalikes”, that have not gained much acceptance on the market. The second product generation comprised e-cigs with more power and refillable liquid tanks. Later e-cig versions gave the option to modify their parts to the likes of the consumer, e.g., by enabling high-power vaporization in combination with low-resistance coils. With pod e-cigs, the product characteristics went in the opposite direction. These products are easy to use as all components other than the battery are disposable and can be bought ready for use. The herein studied pod e-cig has led to a high number of adolescent and young adult consumers in 2019 in the US [15]. Reasons for this were discussed and include the social media marketing strategy, the selection of appealing flavors, product features, and the high nicotine delivery of the US version [16,17,18,19,20]. While we have showed that the European version delivers much less nicotine than a conventional cigarette in a study applying a pre-directed puffing protocol [21], it is unclear what nicotine levels are reached when consumers use the products in their own pattern. It was hypothesized that pod e-cigs may be used differently than known for older generation e-cigs. In their ad libitum study over 90 min, St. Helen et al. found out that e-cig users took puffs with an average duration of 3.5 s. In total, 12% of all puffs were unclustered (one puff within 60 s), 43% were drawn in short clusters (2–5 puffs within 60 s), 28% in medium clusters (6–10 puffs within 60 s), and 17% in long clusters (more than 10 puffs within 60 s) [22]. While puffs drawn in medium or long clusters resemble the consumption behavior of smoking a cigarette, it is hypothesized that pod e-cigs are rather used in an unclustered manner or in short clusters.

In contrast to e-cigs, HTPs contain tobacco that is heated up to temperatures of 350 °C to produce the nicotine-containing aerosol [23]. Recently, we showed that two HTPs reduce craving for the respective product in experienced users, although their nicotine delivery was significantly lower compared with CCs (C_max:_ HTP 1 (the same as used herein): 14.9 mg/mL, HTP 2: 11.6 mg/mL and CC: 25.1 mg/mL) [24]. The question remained whether consumers compensate for the lower nicotine delivery by using the HTP more often than cigarettes would be used.

With the herein presented study, we followed up on our previous study of nicotine delivery in the acute phase during pre-directed use of a pod e-cig [21] and HTPs [24]. The aim of the present study was to gain insights into puffing behavior and nicotine self-titration under near-real-world conditions. The above-mentioned pod e-cig, an HTP, and conventional cigarettes were each consumed by 15 experienced users of the respective product group. During the 90 min study period, participants could use the product ad libitum. They were videorecorded to analyze their puffing behavior. At predetermined time points, blood was sampled to determine plasma nicotine concentrations. Nicotine concentrations were measured using a previously published method for quantifying nicotine and its major metabolites, cotinine and trans-3′-hydroxycotinine, by LC-MS/MS in human plasma [25]. Subjective effects of product use were inquired using questionnaires.

## 2. Materials and Methods

### 2.1. Aims and Ethics

The aim of the present study was to collect information on the addiction potential and addiction satisfaction of pod e-cigs and HTP compared to a conventional tobacco cigarette. For this purpose, subjective effects and nicotine release in the blood were investigated in the acute phase after ad libitum consumption. The study was approved by the Ethics Committee of the LMU Munich (No. 21-0372) and performed in accordance with the principles of the Declaration of Helsinki in the currently valid version. Furthermore, the study was registered at the “Bundesinstitut für Arzneimittel und Medizinprodukte” (BfArM) (at that time “Deutsches Register klinischer Studien” (DRKS)) (DRKS00024751). Informed consent was obtained from all participants prior to the participation in the study.

### 2.2. Study Prodcts and Groups

The study was designed as a single-center, four-arm, open-prospective, explorative study. Products were used ad libitum, meaning that they were used without any specific use instructions such as a puffing protocol. Three products were tested in a parallel group design: A pod e-cig (JUUL, rich tobacco flavor, 18 mg/mL nicotine, “modified version” as described previously [21]), an HTP with 4.7–5.1 mg per cigarette unit (IQOS 3 Duo with their preferred tobacco stick variant) [26,27], and a conventional cigarette. Cigarette users could choose between their own brand filter cigarette and a cigarette provided on test day (Marlboro Red). The nicotine contents of European conventional cigarettes were determined in 2017 and ranged from 9 mg to 17.5 mg per cigarette unit [28]. In a fourth arm, another pod e-cig (myblu, tobacco flavor) was used in a cross-over design with the JUUL e-cig. This part of the study aimed to evaluate whether puffing behavior and nicotine self-titration results are unique for JUUL e-cigs, or whether they have general implications for this e-cig generation. This part of the study will be evaluated and published separately due to statistical reasons.

### 2.3. Participants

Fifteen subjects per product group were recruited between August 2021 and May 2022 via direct, private contacts, internet platforms (especially Facebook), as well as with flyers in e-cig stores. Furthermore, the LMU intranet and the LMU e-mail information service was used for recruitment. Subjects were experienced, dependent users of the product category (CC, e-cigs or HTPs) of the study arm they participated in.

Inclusion criteria were divided into general and specific inclusion criteria. General inclusion criteria were: age of the subjects between 18 and 65 years, 12 h of nicotine abstinence before the investigation (CO in exhaled air less than 5ppm, nicotine plasma concentration at baseline not greater than 10 ng/mL), and ability to give general consent. Specific inclusion criteria were related to the respective test groups. Daily use of the respective product for more than 3 months (for conventional cigarette for more than 5 years) as well as no regular co-use of other nicotine products was required. Exclusion criteria were acute severe psychiatric disease, acute addictive disease, pregnancy, malignant cancer, severe cardiovascular disease, severe infectious disease, and severe pulmonary disease.

All 45 participants were given a detailed verbal explanation of the study aim, implementation, risks, burdens, voluntariness, right of withdrawal, and insurance. All subjects agreed to participate in the study in advanced and gave written consent. Travel and subject insurance were obtained from HDI Global SE for all subjects (Insurance number: 39 130537 03026/03440)

### 2.4. Study Design and Questionnaires

A screening interview was held before the first testing session. Here, subjects were informed about the study, eligibility for participation in the study was determined, and consent for data collection was obtained. Subsequently, socio-demographic data and data on smoking behavior were collected in a preliminary examination according to internally standardized questionnaires. The FTND (Fagerström Test for Nicotine Dependence [29]) was used to assess physical nicotine dependence in the CC group. A modified version of the FTND, which was unvalidated (see Supplementary Material) was used for the pod e-cig and HTP groups.

On the test date, CO in expiratory air was measured using a Smokerlyzer [Bedfond GmbH] before the start of the study, followed by a pregnancy test for women of childbearing age using a contraceptive method with a Pearl index > 0.9.

**Figure 1:**
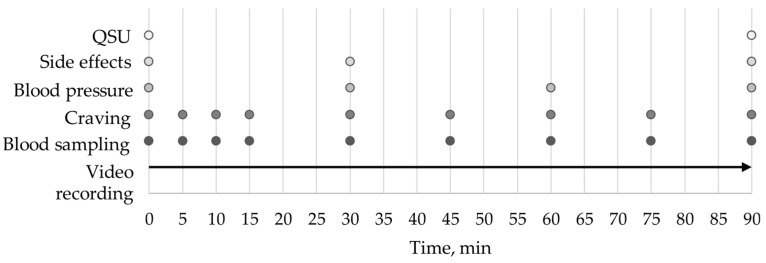
Study design summarizing measurements and their time points.

The test period was 90 min, during which the subjects were free to use their product without any instructions regarding smoking behavior (ad libitum). Measurements at baseline and during the study period of 90 min are summarized in Figure 1.

The German version of the QSU (Questionnaire on Smoking Urges) was used to measure reduction in product use urges at baseline and after completion of the session [30]. This questionnaire measures on two scales the intention to smoke and the expectation of a positive effect of smoking (intention to smoke/positive smoking effect—positive reinforcement scale—Scale 1) as well as the desire to smoke by eliminating withdrawal symptoms (desire to smoke/withdrawal reduction—negative reinforcement scale—Scale 2) [31]. Modified, unvalidated versions of the QSU-G (see Supplementary Material) were used for the pod e-cig and HTP groups.

During the session, subjects were asked to rate their current craving (“I now feel the urge after a JUUL/IQOS/cigarette”) at 5, 10, 15, 30, 45, 60, 75, and 90 min on a scale from 1 (not at all true) to 7 (completely true).

To assess side effects, a standardized internal questionnaire with 12 items (lightheadedness, mouth irritation, throat irritation, dizziness, salivation, cold hands/feet, heart pounding, headache, sweating, nausea, feeling of vomiting, and other) on a scale from 0 (no effect) to 10 (strongest effect) was queried at baseline and min 30 and 90. 

In addition, blood pressure and pulse were measured at 0, 30, 60, and 90 min.

### 2.5. Blood Sampling and Determination of Nictoine, Cotinine, and Hydroxycotinine Plasma Levels

Nicotine kinetics were collected by venous blood sampling through an indwelling venous cannula at baseline and at 5, 10, 15, 30, 45, 60, 75, and 90 min. Blood samples were directly refrigerated, then centrifuged and frozen (−80 °C). The measurement was performed by the German Federal Institute for Risk Assessment (BfR) in Berlin, Germany. The plasma concentrations of nicotine and the metabolites hydroxycotinine and cotinine were analyzed using liquid chromatography coupled with tandem mass spectrometry (LC-MS/MS) following protein precipitation. A matrix-matched calibration was used for quantification. Details of the validated analytical method were previously published [25].

### 2.6. Puffing Behaviour

Usage behavior was recorded by video over the 90 min test period. After the test period, the video was uploaded into a video analysis software (Videograph) and smoking behavior variables were coded. The variables puff duration (duration of lip contact of the product), inhalation time (settling of the product until the first visible aerosol), and exhalation time (first visible aerosol until no more aerosol visible) were coded and rounded to the full second. Topography variables in terms of puffing clusters were coded with clusters being defined according to St. Helen et al.: single puff (puff is not followed by another puff within 60 s), cluster (puff follows a preceding puff within 60 s; short cluster: 2–5 puffs, medium cluster: 6–10 puffs, and long cluster: more than 10 puffs) [22].

### 2.7. Pharmacokinetic (PK) Parameters and Statistical Analysis

The statistical analysis of the data was performed using Excel 2021 and SPSS26. The puff topography data obtained from the video recordings were analyzed with the video analysis software “Videograph” (Version 4.4.2, Rolf Rimmele, Altenholz, Germany). Afterwards, data were exported and processed in Excel 2021 and analyzed with the SPSS26. For this purpose, in addition to *t*-tests for independent samples, Mann–Whitney U-tests were used. Furthermore, Kruskal–Wallis tests were used as an alternative non-parametric method for the analysis of variance. Using the *t*-test, we tested the statistical significance of the QSU-G scores. Median and interquartile ratios (IQR) were calculated for participant characteristics.

Areas under the plasma concentration-time curve (AUC) were calculated with the linear trapezoid rule using baseline-corrected nicotine plasma concentrations (nicotine plasma concentration at baseline was subtracted from following samples). As the C_max_, the highest analyzed nicotine plasma concentration per individual was selected, and for t_max_, the time point of C_max_. Geometric means and coefficients of variance (CV) were calculated for AUC, C_max_, and C_5min_. Two-sided unpaired *t*-tests were used with lognormal values for AUC, and C_max_ values were used for statistical analysis. Mean nicotine plasma curves were built using arithmetic means and 95% confidence intervals. For the statistical analysis of t_max_, median, range, and a two-sided unpaired *t*-test were used. Nicotine metabolic ratio (NMR) can be used as a surrogate marker for CYP 2A6 metabolic activity [32,33]. It was calculated by dividing the baseline plasma concentrations of the metabolites hydroxycotinine by cotinine.

## 3. Results

### 3.1. Participants

Of the 73 subjects recruited, 52 subjects were included after initial screening according to inclusion and exclusion criteria. After 7 drop-outs during the study and post-recruitment, 45 participants (15 per group) were tested. Characteristics of the 45 study participants are summarized in Table 1. 

Across the three arms, the mean age was 26.5 years, ranging from 18 to 49 years. Overall, the sex of the subjects was divided with no significant difference (*p* < 0.914), with 20 females (44%) and 25 males (56%). The overall mean score on the FTND was 4.6 points (“strong physical dependence”).

### 3.2. Usage Behavior

Over the study duration of 90 min, participants were video recorded. Videos were analyzed regarding the number of consumed CCs or HTP sticks if applicable, number and interval of puffs taken, and duration of puffing, inhalation, and exhalation. Puff topography parameters per individual participant are presented in the Supplementary Tables S7–S9. In the HTP and CC groups, the same average number of units were consumed with 4.2 HTP sticks and CCs, respectively. The numbers of puffs taken was significantly different (*p* < 0.041) between all groups (CC 42.3; HTP 52.2 and pod e-cig 71.9). Pairwise comparisons showed a significant difference only between the pod e-cig and CC (*p* < 0.027).

**Figure 2:**
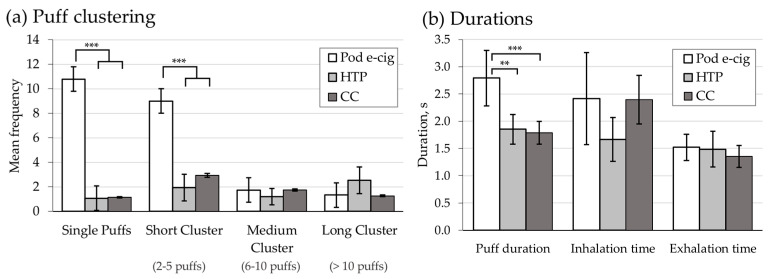
Product use behavior for the three study arms: pod e-cigarette (pod e-cig), heated tobacco products (HTP), and conventional cigarette (CC) with (**a**) mean frequency of single puffs (puff not followed by another puff within 60 s) and different clusters (puff follows a preceding puff within 60 s) with short clusters (2–5 puffs), medium clusters (6–10 puffs), and long cluster (more than 10 puffs) and (**b**) durations of puffing, inhalation and exhalation. Data are presented as mean and coefficient of variance. (** and *** statistically significant ** *p* < 0.01, *** *p* < 0.001).

Clustering of puffs is presented in Figure 2a. Single puffs and short clusters occurred significantly more frequently in the pod e-cig group compared with the HTP group and the CC group (single puff: *p* < 0.001; short clusters *p* < 0.001). No significant difference was observed in the frequency of medium clusters and long clusters between the groups. 

For the length of the puff variables, there was only a significant difference in puff duration between the products (*p* < 0.003) but not in inhalation and exhalation as summarized in Figure 2b. In pairwise comparison, puff duration of the pod e-cig group was significantly longer compared with the HTP (*p* < 0.012) and CC (*p* < 0.006) groups. There was no significant difference in puff duration between the HTP and CC groups.

### 3.3. Nicotine Delivery

Relevant PK parameters, AUC, C_max_, and t_max_, are summarized in Table 2. Overall, nicotine delivery was highest for CC, followed by HTP and subsequently the pod e-cig group. However, differences in geometric mean C_max_ and geometric mean AUC were not statistically significant between the HTP and CC groups. 

**Figure 3:**
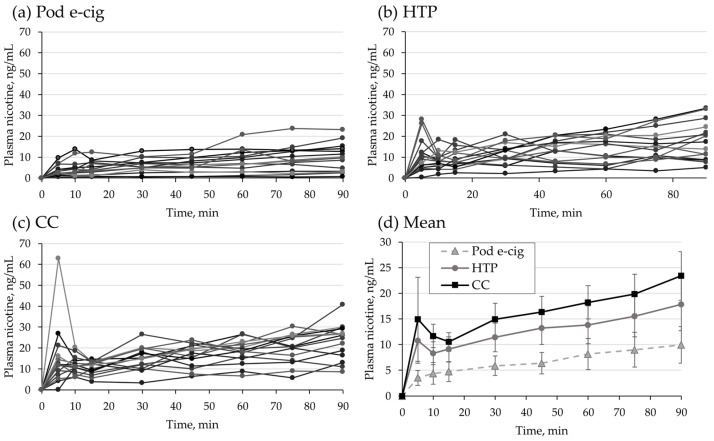
Plasma nicotine curves for the individual participants in (**a**) the pod e-cigarette (pod e-cig) group, (**b**) the heated tobacco product (HTP) group, and (**c**) the conventional cigarette (CC) group. (**d**) Arithmetic means and 95% confidence interval of nicotine plasma curves for the three study arms.

Individual plasma nicotine curves are presented in Figure 3a–c for the pod e-cig group, the HTP group, and the CC group, respectively. Individual concentrations are presented in the Supplementary Tables S1–S3. Figure 3d shows the mean plasma nicotine curves for the three study groups. Repeatedly measured ANOVA shows that the pod e-cig group differs significantly from the other two groups (HTP and CC) over time. In the acute phase, meaning the first 5 min of consumption, geometric means and CV (%) of plasma nicotine concentrations were 2.7 ng/mL (125%), 7.7 ng/mL (180%), and 12.5 ng/mL (97%) for pod e-cig, HTP, and CC, respectively. 

### 3.4. Relief of Craving and Urges to Use the Product

**Figure 4:**
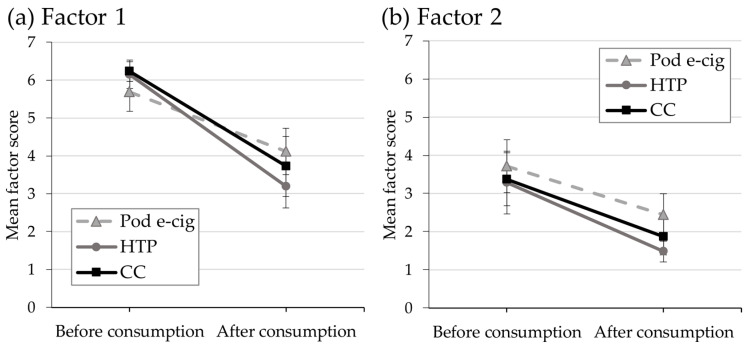
Mean scores for (**a**) factor 1 (positive reinforcement) and (**b**) factor 2 (negative reinforcement) of urges to use the respective study product before and after consumption for the three study arms: pod e-cigarette (pod e-cig) group, heated tobacco products (HTP) group, and conventional cigarette (CC) group. Data are presented as mean and confidence interval.

Urges to use the product were inquired at baseline and after the study period of 90 min using the QSU-G for cigarettes and modified versions for e-cigs or HTPs. Changes in urges to use the products are displayed in Figure 4. Positive reinforcement factors (factor 1, Figure 4a) were statistically significantly reduced over time in all study groups (*p* < 0.001). Between study arms, reduction in urge to use the product was only statistically significantly different between pod e-cig and HTP group (*p* < 0.05). Negative reinforcement factors (factor 2, Figure 4b) were also statistically significantly reduced over time in all study groups (*p* < 0.001). No statistically significant differences between groups were observed.

**Figure 5:**
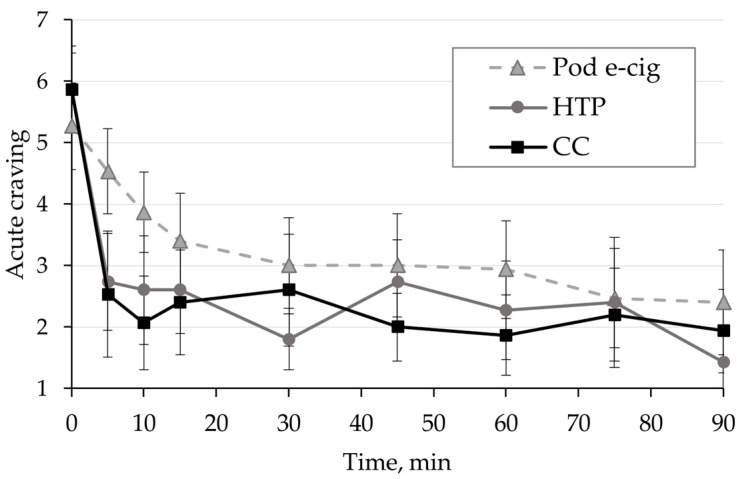
Acute craving for the tested product (single question answered on a scale from 1 to 7) over the study period for the three study arms: pod e-cigarette (pod e-cig) group, heated tobacco products (HTP) group, and conventional cigarette (CC) group. Data are presented as mean and confidence interval.

Acute craving to use the respective study product was inquired at nine time points during the 90 min study period by asking a single question. Mean results are shown in Figure 5 and individual results are presented in the Supplementary Tables S4–S6. A strong, statistically significant reduction of craving over time was observed in all groups (*p* < 0.001). The reduction in craving differed significantly between groups (*p* < 0.012). In the acute phase at 5 min (*p* < 0.001) and 10 min (*p* < 0.001), the reduction in craving was significantly stronger in HTP and CC than in the pod e-cig group. A repeated-measures ANOVA conducted under the assumption of sphericity also shows that all groups change significantly over time, regardless of the product.

### 3.5. Side Effects

Cardiovascular side effects such as changes in heart rate and blood pressure are displayed in Figure S1 in the Supplementary Material. Heart rate of the participants showed a significant increase after 30 min from 71.7 bpm to 78.9 bpm in the CC group (*p* < 0.007) and from 72.5 bpm to 77.1 bpm in the HTP group (*p* < 0.024). For the pod e-cig, there was no significant change after 30 min.

For systolic and diastolic blood pressure, there were no significant changes over time and no significant differences between groups during the 90 min period.

At baseline, after 30 and after 90 min, participants were asked to rate the following side effects from 0 (no effect) to 10 (strong effect): drowsiness, mouth irritation, throat irritation, dizziness, salivation, cold hands/feet, cardiac palpitation, headache, sweating, nausea and feeling of vomiting. Results are summarized in Figure S2 in the Supplementary Material. Overall, side effects were low to moderate.

## 4. Discussion

The aim of the herein presented study was to assess nicotine kinetics and usage behavior during ad libitum use of pod e-cigs and HTPs in comparison with CCs as a near-real-world scenario. For assessment of the users puffing behavior, a method based on video recording was chosen instead of a puff topography device to keep the influence on the users’ puffing behavior as low as possible [34]. 

This study is a follow up of previous work on nicotine kinetics and subjective effects in the acute phase of product use. Following a pre-directed puffing regime, experienced users have used either a pod e-cig [26] o HTPs [24].

After pre-directed consumption of a pod e-cig, a maximum plasma nicotine concentration of 6.3 ng/mL was reached with a t_max_ of 6 min [26]. After the first 5 min of the 90 min ad libitum use of the same pod e-cig in the herein presented study, only 2.7 ng/mL was reached. After 90 min, the maximum plasma nicotine level of 8.0 ng/mL was measured. The experienced e-cig users did not titrate their plasma nicotine levels to concentrations that are comparable to CCs. In the CC control groups, a C_max_ of 14.4 ng/mL was reached after 8 min in the first study [26] and a plasma concentration of 12.5 ng/mL was reached after 5 min in the present study. In a study on the US version of the pod e-cig (approx. 58 mg/mL nicotine), smokers without e-cig experience had a mean plasma nicotine concentration of 11.5 ng/mL after 90 min ad libitum use [35]. Mean C_max_ during 90 min ad libitum use of different generation, own-brand e-cigs was 12.8 ng/mL with a median t_max_ of 90 min [22].

Regarding the puff behavior during e-cig use, various studies have been conducted [22,36,37,38]. For example, analysis of internet videos has revealed that e-cig users draw significantly longer puffs compared with smokers of CCs [37]. In a review from 2018, it was summarized that e-cigs differ in their puff behavior compared to CC users—they take longer puffs and have longer use bouts [38]. Experienced e-cig users draw longer puffs compared with unexperienced users, whereas their inhalation time is 1.3 ± 0.4 s lower compared to smokers, who do not usually smoke e-cigs (2.0 ± 0.4) [39]. In an ad libitum study published in 2016 on e-cigs of different generations, the mean puffing duration of one puff was 3.5 ± 1.4 s and the interval between puffs was 118 ± 141 [22]. The mean frequencies of the different clusters were 5.2 for single puff, 8.6 for short cluster (2–5 puffs), 2.4 for medium cluster (6–10 puffs), and 0.8 for long puffs (>10 puffs) [22].

In line with previous findings, pod e-cigs were used with the longest puff duration (2.8 s) compared to HTP (1.9 s) and CC (1.8 s) in the present study. During the 90 min study period, single puffs occurred most often followed by short clusters with mean frequencies of 10.8 and 9.0, respectively. Medium and long clusters were rare in the pod e-cig group with mean frequencies of 1.7 and 1.3, respectively. The patterns of e-cig use in both studies, St. Helen et al. [22] and the presented one, differed significantly from CC or HTP use. For HTPs and CCs, units are consumed that require a clustering of several puffs over a short time period. As the consumption unit is “burned off”, product is wasted otherwise. The product design of e-cigs on the other hand allows for shorter clusters without wasting product. In the present study, a newer pod e-cig containing nicotine salt and a higher nicotine concentration (18 mg/mL) was used compared with the previous study [22], showing a slightly different usage pattern towards a higher frequency of single puffs. This might be due to different design features. For example, the lower pH of nicotine salt-containing e-cig liquids reduces the amount of free-base nicotine-enabling inhalation of high nicotine concentrations with lower adverse sensory effects [40,41,42]. In a machine-vaping experiment, the pod e-cig emitted a high amount of nicotine per puff with 61 µg/puff [26]. Thus, it is possible that single puffs can deliver a high enough bolus of nicotine during intermittent self-titration. 

In the previous study on HTPs, a C_max_ of 14.9 ng/mL was obtained after experienced users took ten pre-directed puffs with a t_max_ of 6 min [24]. In the present study, a plasma nicotine concentration of 7.7 ng/mL was reached after the first 5 min of ad libitum use of the same HTP. However, after 90 min of HTP use, the mean plasma nicotine concentration was 17.7 ng/mL. In contrast to the previous study [24], C_max_ and AUC did not differ significantly between the HTP and CC groups in this study. Although it should be noted that different participants took part in the studies, the results still suggest that during ad libitum consumption over 90 min, HTP users extract a considerable amount of nicotine from their products. Smokers who were inexperienced with HTP use had a mean plasma nicotine concentration of 11.3 ng/mL after 90 min ad libitum use in a previous study [35], lower compared to the herein presented results in experienced users. The manufacturer of the HTP conducted a study with 24 h ad libitum use by CC smokers and reported a mean peak nicotine concentrations of 14.9 ng/mL and 24.0 ng/mL for the HTP and for CC, respectively [43].

Due to the previously reported lower nicotine delivery after consumption of a single HTP stick in comparison with a single CC, the question arose whether this leads to a consumption of more HTP consumption units [24]. In the past, it has been shown that a compensatory increase in product use was triggered by low yield cigarettes [44]. In the present study, the same amount of HTP sticks and CC were used over the 90 min study period. A compensatory increase in product use was not observed. However, 90 min is a rather short time period and compensatory product use may still occur over longer periods. In studies by the manufacturer, it was shown that the number of consumption units was higher for the HTP groups compared with the CC groups over testing periods of 5 days [45,46].

It is noteworthy that the successful reduction of craving and reduction of positive and negative reinforcement factors for product use occurred for all three product uses; despite the differences in delivered nicotine levels. In the acute phase, CC and HTP users had a faster rise of plasma nicotine levels and a faster craving reduction. In the pod e-cig group, plasma nicotine curves do not show an initial peak in the acute phase. Consequently, craving reduction was the slowest in the pod e-cig group. However, craving was reduced in all products to similar levels at the end of the study period.

At the physiological level, the maximum achieved concentration of nicotine levels does not seem to be the decisive value for successful craving reduction in the comparison among the products. In our study, even lower nicotine levels than those achieved with CCs can be accompanied by a sufficient reduction in craving for the dependent user. In the literature, there are no distinct plasma levels of nicotine that define dependence. In particular, the nicotine plasma levels achieved with standardized tobacco products such as the filter cigarette are known. Numerous studies with different methodological approaches have been conducted; they came to the conclusion that nicotine accumulation in tobacco smoking depends on various individual preconditions as well as product preconditions. Factors such as habitual smoking, enzyme induction or genetic variability can influence nicotine concentrations as well as the brand of the cigarette and individual smoking behavior. This underlines the assessment that there are no clear plasma levels for dependence, but that dependence is multifactorial [47,48,49,50]. Whether these are absolutely necessary for the development and maintenance of dependence or whether lower maximum concentrations are already sufficient remains unclear.

As a behavioral aspect, the difference between fixed units of consumption (HTP and CC) and continuous use (pod e-cig) could play a role in craving. For each CC and HTP consumption, preparation is required (taking the unit of use out of the pack, activating the device/lighting the CC), while preparation for pod e-cig use is minimal.

Limiting factors in this study are the clinical setting and the rather short observation period of 90 min as well as the small number of probands (15 per group). The daily course of use of the different products is not reflected with this study design. Thus, it remains open which plasma nicotine levels the pod e-cig users would have achieved after longer use and whether they experience withdrawal due to plasma nicotine troughs.

It should be noted that direct comparisons between results obtained with the different study arms are limited since there was no cross-over. The parallel-group design of the study was necessary to include experienced and exclusive users of the respective products. Information on puff volume is missing as they cannot be extracted with the method chosen for puff recording. However, as discussed above, a mouth-piece device could have affected other topography parameters. No validated German versions of FTND and QSU exist for e-cigs and HTPs. Thus, unvalidated versions had to be used in both study arms. Furthermore, in this clinical setting, only a near-real-world scenario can be represented. In the future, more field studies are needed to elucidate usage behavior and titration of plasma nicotine levels with ENDS.

## Figures and Tables

**Table 1:** Participant characteristics per study arm: pod e-cigarette (pod e-cig) group, heated tobacco products (HTP) group, and conventional cigarette (CC) group. Age, score of modified Fagerstrom Test for Nicotine Dependence (FTND), nicotine metabolic ratio (NMR), and days of product use in the past 30 days are presented as median with interquartile interval.

	Pod E-Cig Group	HTP Group	CC Group
Recruited	19	24	30
Included	16	18	18
Drop-out	1	3	3
Tested	15	15	15
Age	25.7 (9.5)	27.1 (8.5)	26.7 (7.5)
Sex, female, n (%)	6 (40)	7 (47)	7 (47)
Sex, male, n (%)	9 (60)	8 (53)	8 (53)
FTND	5.2 * (3.5)	4.4 * (1)	4.3 (2.5)
NMR	0.38 (0.19)	0.38 (0.53)	0.36 (0.54)
Days of product use in past 30 days	29.7 (0)	29.9 (0)	29.1 (0)

* FTND modified for e-cigs or HTP.

**Table 2:** Summary of relevant PK parameters and statistical evaluation for the three study arms: pod e-cigarette (pod e-cig) group, heated tobacco products (HTP) group, and conventional cigarette (CC) group.

	Pod E-Cig	HTP	CC	Pod E-Cig vs. HTP	Pod E-Cig vs. CC	HTP vs. CC
C_max_ (ng/mL)	8.0 (136%)	17.7 (66%)	24.0 (45%)	*p* = 0.018 **	*p* < 0.001 ***	*p* = 0.130
AUC_0–90min_ (ng/mL * h)	8.3 (126%)	17.3 (68%)	24.8 (51%)	*p* = 0.014 **	*p* < 0.001 ***	*p* = 0.226
t_max_ (min)	90 (5–90)	75 (5–90)	75 (5–90)	*p* = 2.2	*p* = 1.1	*p* = 1.5

C_max_ and AUC: Geometric mean and coefficient of variance (CV%), *p*-values obtained with Bonferroni corrected unpaired, two-sided *t*-test with lognormal values; t_max_: Median and range, *p*-values obtained with Bonferroni corrected unpaired, two-sided *t*-test; *, ** and *** statistically significant (* *p* < 0.05, ** *p* < 0.01, *** *p* < 0.001).

## Data Availability

The data presented in this study are available in the Supplementary Material.

## Supplementary Materials

The following supporting information can be downloaded at: https://www.mdpi.com/article/10.3390/toxics11050434/s1, Modified questionnaires FTND, QSU-G; Tables S1–S3, Nicotine plasma concentrations, Tables S4–S6, Acute craving; Tables S7–S9, Puff topography; Figures S1 and S2, side effects.
