# Supplementary material for: Usage Pattern and Nicotine Delivery during Ad Libitum Consumption of Pod E-Cigarettes and Heated Tobacco Products
Source: Toxics. 2023 May 5;11(5):434. doi: 10.3390/toxics11050434 (PMC10221897; doi:10.3390/toxics11050434)
Supplement: Supplementary file 1 [file toxics-11-00434-s001.zip › toxics-2328775-supplementary.pdf]

Supplementary Material

Usage Pattern and Nicotine Delivery during Ad Libitum Consumption of Pod E-Cigarettes and Heated Tobacco Products

Andrea Rabenstein [1,*,†], Anna Rahofer [1,†], Jochen Vukas [1], Benedikt Rieder [1], Kristin Störzenhofecker [2], Yvonne Stoll [1], Nestor Burgmann [1], Elke Pieper [3], Peter Laux [3], Andreas Luch [3], Tobias Rüther [1,‡] and Nadja Mallock-Ohnesorg [3,‡]

[1]Department of Psychiatry and Psychotherapy, University Hospital, Ludwig Maximilian University of Munich (LMU), Nussbaumstraße 7, 80336 Munich, Germany

[2]Department of Social Services, Katholische Hochschule Nordrhein-Westfalen, Standort Köln, Wörthstraße 10, 50668 Cologne, Germany

[3]German Federal Institute for Risk Assessment (BfR), Department of Chemical and Product Safety, Max-Dohrn-Straße 8-10, 10589 Berlin, Germany

†These authors contributed equally to this work.

‡These authors contributed equally to this work.

*Correspondence: andrea.rabenstein@med.uni-muenchen.de

Table of Contents

1. Modified Questionnaires 2
   1.1. Modified FTND 2
   1.2. Modified QSU-G 3
2. Nicotine Plasma Concentrations of Individual Participants 8
3. Craving 10
4. Side Effects 12

1. Modified Questionnaires

Modified versions of Fagerstrom Test of Nicotine Dependence (FTND) and Questionnaire on Smoking Urges (QSU-G) in German were used to fit the research question as presented below. Rephrasing of items was kept at a minimum, scoring and interpretation remained the same.

1.1. Modified FTND

a. Modified FTND for heated tobacco products:

1. Wann nach dem Aufwachen konsumieren Sie Ihren ersten Stick?
   - Innerhalb von 5 Minuten (3)
   - Nach 6-30 Minuten (2)
   - Nach 31-60 Minuten (1)
   - Nach 60 Minuten (0)
2. Finden Sie es schwierig, auf die IQOS zu verzichten, wenn es verboten ist? (z. B. im Kino, in Versammlungen usw.)
   - Ja (1)
   - Nein (0)
3. Auf welchen Stick würden Sie nicht verzichten wollen?
   - Den 1. Stick morgens (1)
   - Jeden anderen Stick (0)
4. Wie viele Sticks konsumieren Sie am Tag?
   - 0-10 (0)
   - 11-20 (1)
   - 21-30 (2)
   - 31 und mehr (3)
5. „Rauchen“ bzw. konsumieren Sie am Morgen im Allgemeinen mehr als am Rest des Tages?
   - Ja (1)
   - Nein (0)
6. Kommt es vor, dass Sie IQOS nutzen, wenn Sie krank sind und tagsüber im Bett bleiben müssen?
   - Ja (1)
   - Nein (0)

Gesamtpunktzahl: [__I__]

FTND: 0-2 geringe, 3-4 mittlere, 5-6 starke, 7-10 sehr starke körperliche Abhängigkeit

HSI: 0-2 geringe, 3-4 mittlere, 5-6 starke körperliche Abhängigkeit

b. Modified FTND for pod e-cigarettes

1. Wie schnell nach dem Aufwachen dampfen Sie zum ersten Mal?
   - innerhalb von 5 Minuten (3)
   - 6-30 Minuten (2)
   - 31-60 Minuten (1)
   - nach 60 Minuten (0)
2. Finden Sie es schwierig, auf das Dampfen zu verzichten, wenn es verboten ist?
   - Ja (1)
   - Nein (0)
3. Wann fällt es Ihnen besonders schwer auf das Dampfen zu verzichten?
   - Das erste Mal dampfen am Morgen (1)
   - jedes andere Mal (0)
4. Wie viele Pods (18mg/ml Nikotin | 0,7ml | 12,6mg Nikotin) dampfen Sie am Tag?
   - 0 - 0,5 (0)
   - 0,5 - 1 (1)
   - 1 - 1,5 (2)
   - 1,5 und mehr (3)
5. Dampfen Sie stärker in den ersten Stunden nach dem Aufstehen als während des übrigen Tages?
   - Ja (1)
   - Nein (0)
6. Dampfen Sie auch, wenn Sie so krank sind, dass Sie im Bett liegen müssen?
   - Ja (1)
   - Nein (0)

Gesamtpunktzahl: [_I_]

FTND: 0-2 geringe, 3-4 mittlere, 5-6 starke, 7-10 sehr starke körperliche Abhängigkeit

HSI: 0-2 geringe, 3-4 mittlere, 5-6 starke körperliche Abhängigkeit

1.2. Modified QSU-G

a. Modified QSU-G for heated tobacco products:

Bitte kennzeichnen Sie die für Sie zutreffende Einschätzung mit einem Kreis.
Bitte achte Sie auf Verneinungen durch das Wort „nicht“.

	Stimmt Überhaupt nicht						stimmt völlig
1. Eine IQOS zu nutzen würde mir jetzt dazu verhelfen, mich sehr gut zu fühlen.	1	2	3	4	5	6	7

2. Ich wäre weniger reizbar, wenn ich jetzt eine IQOS nutzen könnte.	1	2	3	4	5	6	7
3. Nichts wäre besser, als jetzt eine IQOS zu nutzen.	1	2	3	4	5	6	7
4. Mir fehlt die IQOS jetzt gerade ***nicht***.	1	2	3	4	5	6	7
5. Ich werde eine IQOS nutzen, sobald ich wieder die Möglichkeit dazu bekomme.	1	2	3	4	5	6	7
6. Ich möchte jetzt ***nicht*** IQOS nutzen.	1	2	3	4	5	6	7
7. Eine IQOS würde meine schlechte Stimmung deutlich verbessern.	1	2	3	4	5	6	7
8. Eine IQOS würde mir jetzt ***nicht*** helfen, mich zu beruhigen.	1	2	3	4	5	6	7
9. Wenn ich jetzt eine IQOS angeboten bekomme, würde ich sie sofort verwenden.	1	2	3	4	5	6	7
10. Ich könnte ab sofort für eine lange Zeit auf das Verwenden von IQOS verzichten.	1	2	3	4	5	6	7
11. Jetzt eine IQOS zu nutzen, wäre ***nicht*** angenehm.	1	2	3	4	5	6	7
12. Wenn ich jetzt im Moment eine IQOS würde nutzen, wäre ich weniger gelangweilt.	1	2	3	4	5	6	7
13. Das Einzige, was ich jetzt gerne hätte, wäre eine IQOS.	1	2	3	4	5	6	7
14. Wenn ich jetzt eine IQOS nutzen würde, würde ich mich weniger müde fühlen.	1	2	3	4	5	6	7
15. Jetzt einen Stick zu konsumieren würde mich glücklicher machen.	1	2	3	4	5	6	7
16. Sogar, wenn es jetzt möglich wäre, würde ich IQOS wahrscheinlich ***nicht*** verwenden.	1	2	3	4	5	6	7

17.	Ich habe jetzt gerade keinen Wunsch nach IQOS.	1	2	3	4	5	6	7
18.	Mein Wunsch nach einer IQOS scheint gerade so stark zu sein, dass er mich zu überwältigen droht.	1	2	3	4	5	6	7
19.	Wenn ich jetzt eine IQOS nutzen würde, erschiene mir fast alles in Ordnung.	1	2	3	4	5	6	7
20.	Ich sehne mich gerade nach einer IQOS.	1	2	3	4	5	6	7
21.	Ich würde eine IQOS jetzt ***nicht*** genießen.	1	2	3	4	5	6	7
22.	Eine IQOS würde jetzt ***nicht*** gut schmecken.	1	2	3	4	5	6	7
23.	Ich verspüre jetzt gerade den Drang nach einer IQOS.	1	2	3	4	5	6	7
24.	Ich hätte die Dinge gerade besser im Griff, wenn ich eine IQOS nutzen würde.	1	2	3	4	5	6	7
25.	Ich werde eine IQOS nutzen, sobald ich die Möglichkeit dazu habe.	1	2	3	4	5	6	7
26.	Ich würde mich körperlich ***nicht*** besser fühlen, wenn ich jetzt einen Stick konsumieren würde.	1	2	3	4	5	6	7
27.	Eine IQOS wäre jetzt ***nicht*** sehr befriedigend.	1	2	3	4	5	6	7
28.	Wenn ich jetzt eine IQOS in der Hand hätte, würde ich sie wahrscheinlich gar ***nicht*** nutzen.	1	2	3	4	5	6	7
29.	Wenn ich jetzt einen Stick konsumieren würde, könnte ich klarer denken.	1	2	3	4	5	6	7
30.	Ich würde fast alles tun, um jetzt eine IQOS zu bekommen.	1	2	3	4	5	6	7
31.	Ich muss jetzt einen Stick konsumieren.	1	2	3	4	5	6	7
32.	Gerade jetzt habe ich ***nicht*** vor eine IQOS zu nutzen.	1	2	3	4	5	6	7

a. Modified QSU-G for pod e-cigarettes

Bitte kennzeichnen Sie die für Sie zutreffende Einschätzung mit einem Kreis.
Bitte achte Sie auf Verneinungen durch das Wort „nicht“.

	Stimmt Überhaupt nicht						stimmt völlig
1. Eine E-Zigarette zu dampfen würde mir jetzt dazu verhelfen, mich sehr gut zu fühlen.	1	2	3	4	5	6	7
2. Ich wäre weniger reizbar, wenn ich jetzt eine E-Zigarette dampfen könnte.	1	2	3	4	5	6	7
3. Nichts wäre besser, als jetzt eine E-Zigarette zu dampfen.	1	2	3	4	5	6	7
4. Mir fehlt das Dampfen jetzt gerade ***nicht***.	1	2	3	4	5	6	7
5. Ich werde eine E-Zigarette dampfen, sobald ich wieder die Möglichkeit dazu bekomme.	1	2	3	4	5	6	7
6. Ich möchte jetzt ***nicht*** dampfen.	1	2	3	4	5	6	7
7. Eine E-Zigarette würde meine schlechte Stimmung deutlich verbessern.	1	2	3	4	5	6	7
8. Eine E-Zigarette würde mir jetzt ***nicht*** helfen, mich zu beruhigen.	1	2	3	4	5	6	7
9. Wenn ich jetzt eine E-Zigarette angeboten bekomme, würde ich sie sofort dampfen.	1	2	3	4	5	6	7
10. Ich könnte ab sofort für eine lange Zeit auf das Dampfen verzichten.	1	2	3	4	5	6	7
11. Jetzt eine E-Zigarette zu dampfen, wäre ***nicht*** angenehm.	1	2	3	4	5	6	7
12. Wenn ich jetzt im Moment dampfen würde, wäre ich weniger gelangweilt.	1	2	3	4	5	6	7

13. Das Einzige, was ich jetzt gerne hätte, wäre eine E-Zigarette.	1	2	3	4	5	6	7
14. Wenn ich jetzt eine E-Zigarette dampfen würde, würde ich mich weniger müde fühlen.	1	2	3	4	5	6	7
15. Jetzt zu dampfen, würde mich glücklicher machen.	1	2	3	4	5	6	7
16. Sogar, wenn es jetzt möglich wäre, würde ich wahrscheinlich ***nicht*** dampfen.	1	2	3	4	5	6	7
17. Ich habe jetzt gerade keinen Wunsch nach einer E-Zigarette.	1	2	3	4	5	6	7
18. Mein Wunsch nach einer E-Zigarette scheint geradeso stark zu sein, dass er mich zu überwältigen droht.	1	2	3	4	5	6	7
19. Wenn ich jetzt eine E-Zigarette dampfen würde, erschiene mir fast alles in Ordnung.	1	2	3	4	5	6	7
20. Ich sehne mich gerade nach einer E-Zigarette.	1	2	3	4	5	6	7
21. Ich würde eine E-Zigarette jetzt ***nicht*** genießen.	1	2	3	4	5	6	7
22. Eine E-Zigarette würde jetzt ***nicht*** gut schmecken.	1	2	3	4	5	6	7
23. Ich verspüre jetzt gerade den Drang nach einer E-Zigarette.	1	2	3	4	5	6	7
24. Ich hätte die Dinge gerade besser im Griff, wenn ich eine E-Zigarette dampfen würde.	1	2	3	4	5	6	7
25. Ich werde eine E-Zigarette dampfen, sobald ich die Möglichkeit dazu habe.	1	2	3	4	5	6	7
26. Ich würde mich körperlich ***nicht*** besser fühlen, wenn ich jetzt dampfen würde.	1	2	3	4	5	6	7

27. Eine E-Zigarette wäre jetzt ***nicht*** sehr befriedigend. 1 2 3 4 5 6 7

28. Wenn ich jetzt eine E-Zigarette in der Hand hätte, würde ich sie wahrscheinlich gar ***nicht*** dampfen. 1 2 3 4 5 6 7

29. Wenn ich jetzt eine dampfen würde, könnte ich klarer denken. 1 2 3 4 5 6 7

30. Ich würde fast alles tun, um jetzt eine E-Zigarette zu bekommen. 1 2 3 4 5 6 7

31. Ich muss jetzt eine E-Zigarette dampfen. 1 2 3 4 5 6 7

32. Gerade jetzt habe ich ***nicht*** vor zu dampfen. 1 2 3 4 5 6 7

2. Nicotine Plasma Concentrations of Individual Participants

Nicotine plasma concentrations at the different time points are listed in Tables S1 (for the pod e-cigarette group), S2 (for the HTP group), and S3 (for the conventional cigarette group).

Table S1. Nicotine plasma concentrations of individual participants before baseline correction in the pod e-cigarette group with their nicotine metabolic ratios (NMR).

Participant	NMR	0 min	5 min	10 min	15 min	30 min	45 min	60 min	75 min	90 min
Pod e-cig 1	0.33	0.9	5.0	6.0	6.2	8.3	10.8	13.2	14.1	13.7
Pod e-cig 2	0.47	0.4	1.7	3.1	3.4	5.4	8.6	10.3	13.3	15.7
Pod e-cig 3	0.42	0.5	3.9	5.0	4.2	7.4	6.9	7.4	7.1	5.3
Pod e-cig 4	0.38	0.3	1.4	2.9	7.3	5.5	5.7	6.9	8.8	10.6
Pod e-cig 5	0.64	0.7	10.4	14.5	9.2	13.7	14.3	14.6	13.9	14.7
Pod e-cig 6	0.38	0.8	5.0	5.0	4.3	5.7	7.5	7.4	9.0	11.1
Pod e-cig 7	0.27	0.9	8.0	12.8	13.4	11.2	12.4	21.7	24.8	24.2
Pod e-cig 8	0.79	1.5	3.3	4.5	5.6	11.4	11.2	11.8	16.3	20.7
Pod e-cig 9	0.20	0.0	6.8	6.6	7.4	5.3	5.4	4.7	7.6	8.7
Pod e-cig 10	0.00	0.0	0.5	0.6	0.6	0.7	0.8	1.3	1.7	2.5
Pod e-cig 11	0.45	0.3	4.5	4.7	8.5	7.7	7.6	9.2	10.8	11.7
Pod e-cig 12	0.23	0.2	1.5	1.3	1.1	0.8	0.9	0.9	0.8	0.8
Pod e-cig 13	0.46	0.3	2.6	1.6	1.7	2.8	3.1	3.2	3.5	3.6
Pod e-cig 14	0.33	0.2	2.8	1.2	1.2	4.1	2.9	3.2	2.4	3.3
Pod e-cig 15	0.37	0.5	2.8	3.3	3.8	5.4	5.2	14.1	8.5	10.2

Table S2. Nicotine plasma concentrations of individual participants before baseline correction in the heated tobacco product group with their nicotine metabolic ratios (NMR).

Participant	NMR	0 min	5 min	10 min	15 min	30 min	45 min	60 min	75 min	90 min
HTP 1	0.17	1.7	14.0	10.1	9.1	7.5	14.9	12.3	11.3	10.3
HTP 2	0.19	1.6	6.7	7.4	8.3	10.9	8.8	7.6	12.3	10.3
HTP 3	0.38	0.3	9.4	8.4	8.0	6.4	5.6	4.7	10.4	7.9
HTP 4	0.40	0.3	11.2	8.4	12.4	9.7	13.5	10.7	16.1	11.7
HTP 5	0.55	0.7	5.5	4.8	4.9	10.5	8.2	7.3	9.5	12.2
HTP 6	0.73	0.0	6.5	6.1	5.7	9.2	16.8	16.0	14.8	14.0
HTP 7	0.39	0.3	26.5	8.5	7.7	13.7	8.3	10.1	10.1	20.5
HTP 8	0.32	1.0	18.7	10.6	19.2	10.1	18.8	22.9	26.1	29.8
HTP 9	0.86	0.4	12.4	18.9	16.1	13.9	20.9	21.4	18.9	21.3
HTP 10	0.73	0.6	10.6	10.7	13.9	21.6	13.3	17.1	14.0	22.5
HTP 11	0.14	0.5	4.6	4.8	9.6	14.5	17.4	18.0	16.9	17.9
HTP 12	0.21	1.6	7.9	8.7	6.8	14.7	22.0	24.9	29.8	35.1
HTP 13	0.05	3.0	3.4	4.9	5.6	5.2	6.2	7.4	6.5	8.2
HTP 14	0.23	3.5	10.9	16.6	15.9	20.6	18.7	23.7	23.9	28.0
HTP 15	1.35	0.0	28.2	11.0	8.5	17.9	20.3	18.9	27.4	33.1

Table S3. Nicotine plasma concentrations of individual participants before baseline correction in the conventional cigarette group with their nicotine metabolic ratios (NMR).

Participant	NMR	0 min	5 min	10 min	15 min	30 min	45 min	60 min	75 min	90 min
CC 1	0.36	1.8	28.6	15.6	16.4	16.9	22.9	28.2	22.4	27.8
CC 2	0.75	1.2	5.4	7.3	5.0	4.4	7.5	10.0	6.9	14.2
CC 3	0.25	0.8	7.9	11.5	9.1	13.1	16.3	27.6	21.0	25.5
CC 4	0.22	1.0	13.8	8.4	6.7	11.1	8.5	7.5	9.9	9.7
CC 5	0.87	0.0	14.4	12.6	12.3	19.9	15.9	22.4	30.3	26.5
CC 6	0.86	1.3	64.1	21.6	14.7	21.5	20.0	21.6	27.7	31.3
CC 7	0.37	1.3	6.6	7.3	11.5	16.7	21.2	23.0	26.6	28.1
CC 8	0.15	1.6	10.8	10.2	8.6	13.6	11.5	17.5	15.5	12.5
CC 9	0.19	0.9	22.0	19.6	14.3	27.5	23.2	20.5	21.5	30.9
CC 10	0.87	0.3	9.2	15.6	13.8	10.4	21.8	18.1	25.0	41.0
CC 11	0.27	0.1	11.8	10.7	9.0	18.2	11.6	12.5	13.3	19.0
CC 12	0.31	0.2	12.3	12.1	9.6	17.6	15.1	18.8	26.1	29.6
CC 13	0.59	0.0	0.0	11.1	12.3	9.2	17.5	14.9	20.0	16.5
CC 14	0.15	3.1	19.2	14.2	15.0	17.9	22.5	26.1	29.0	30.0
CC 15	0.38	0.2	12.1	11.2	13.3	19.9	24.1	18.5	16.7	22.4

3. Craving

Acute craving at the different time points are listed in Tables S4 (for the pod e-cigarette group), S5 (for the HTP group), and S6 (for the conventional cigarette group).

Table S4. Acute craving in the pod e-cigarette group (single question answered on a scale from 1 to 7) over the study period per participant.

Participant	0 min	5 min	10 min	15 min	30 min	45 min	60 min	75 min	90 min
Pod e-cig 1	5	6	6	5	5	4	3	1	1
Pod e-cig 2	4	4	4	4	4	5	4	5	5
Pod e-cig 3	7	7	4	2	2	1	1	1	1
Pod e-cig 4	4	4	3	2	2	2	2	2	1
Pod e-cig 5	6	5	5	5	5	4	4	3	2
Pod e-cig 6	6	4	4	1	1	1	1	1	1
Pod e-cig 7	5	3	3	3	3	3	2	2	2
Pod e-cig 8	6	4	3	2	1	1	1	1	1
Pod e-cig 9	7	4	3	3	1	2	2	1	2
Pod e-cig 10	5	5	3	4	3	2	4	2	2
Pod e-cig 11	2	2	2	2	2	2	2	2	2
Pod e-cig 12	5	5	6	6	4	4	5	4	3
Pod e-cig 13	6	6	4	4	4	5	5	5	6
Pod e-cig 14	5	5	5	4	4	4	4	4	4
Pod e-cig 15	6	4	3	4	4	5	4	3	3
Mean	5,3	4,5	3,9	3,4	3,0	3,0	2,9	2,5	2,4

Table S5. Acute craving in the HTP group (single question answered on a scale from 1 to 7) over the study period per participant.

Participant	0 min	5 min	10 min	15 min	30 min	45 min	60 min	75 min	90 min
HTP 1	4	2	1	1	1	1	1	1	1
HTP 2	7	3	5	4	1	2	5	1	2
HTP 3	7	3	1	1	2	4	5	1	1
HTP 4	3	3	3	3	2	1	3	1	2
HTP 5	6	2	2	3	1	2	4	7	3
HTP 6	5	1	3	3	1	5	1	1	1
HTP 7	7	2	2	3	1	3	1	2	1
HTP 8	7	3	6	5	3	2	1	2	1
HTP 9	7	2	1	1	3	2	1	2	1
HTP 10	7	5	4	3	2	4	3	3	x
HTP 11	7	6	5	2	1	4	1	2	3
HTP 12	6	1	1	2	1	2	2	1	1
HTP 13	5	1	1	1	3	1	2	5	1
HTP 14	6	6	2	4	2	4	2	5	1
HTP 15	4	1	2	3	3	4	2	2	1
Mean	5,9	2,7	2,6	2,6	1,8	2,7	2,3	2,4	1,4

Table S6. Acute craving in the cigarette group (single question answered on a scale from 1 to 7) over the study period per participant.

Participant	0 min	5 min	10 min	15 min	30 min	45 min	60 min	75 min	90 min
CC 1	6	4	4	1	5	4	2	4	4
CC 2	6	2	2	2	4	2	2	3	1
CC 3	3	6	1	3	2	2	1	4	2
CC 4	7	1	1	1	1	1	2	1	1
CC 5	7	1	3	3	1	1	1	5	2
CC 6	7	4	3	2	2	3	2	2	2
CC 7	6	4	5	5	2	3	3	3	1
CC 8	7	1	1	1	1	2	1	1	1
CC 9	6	1	1	3	2	3	1	2	3
CC 10	6	1	2	3	4	2	3	1	2
CC 11	5	1	1	3	1	3	1	1	2
CC 12	5	2	1	1	1	1	1	1	1
CC 13	5	6	1	1	3	1	5	3	5
CC 14	6	1	1	1	6	1	1	1	1
CC 15	6	3	4	6	4	1	1	1	1
Mean	5,9	2,5	2,1	2,4	2,6	2,0	1,9	2,2	1,9

4. Puff topography

Acute craving at the different time points are listed in Tables S7 (for the pod e-cigarette group), S8 (for the HTP group), and S9 (for the conventional cigarette group).

Table S7. Puff topography in the pod e-cigarette group.

Participant	Median Mouth time (sec)	Median Inhalationtime (sec)	Median Exhalationtime (sec)	puffs	Single puff	Short cluster	Medium cluster	Long cluster
Pod e-cig 1	02,962	04,765	01,459	52	1	16	1	0
Pod e-cig 2	02,629	01,040	01,742	124	7	9	4	4
Pod e-cig 3	02,980	01,000	01,180	50	6	13	2	0
Pod e-cig 4	02,733	01,567	02,467	30	19	4	0	0
Pod e-cig 5	04,254	02,068	02,254	59	24	8	1	1
Pod e-cig 6	04,333	05,296	01,296	27	21	3	0	0
Pod e-cig 7	03,661	01,964	01,384	112	8	6	1	4
Pod e-cig 8	02,773	02,443	01,216	88	9	10	6	0
Pod e-cig 9	01,747	02,316	01,924	83	10	10	2	1
Pod e-cig 10	01,209	02,364	01,209	110	2	12	3	3
Pod e-cig 11	03,489	05,378	01,356	45	12	10	1	0
Pod e-cig 12	01,655	01,500	01,338	148	0	1	2	7
Pod e-cig 13	02,902	01,000	01,024	41	11	10	0	0
Pod e-cig 14	01,714	02,490	01,837	49	18	13	0	0
Pod e-cig 15	02,820	01,033	01,115	61	14	10	3	0

Table S8. Puff topography in the HTP group.

Participant	Median Mouth time (sec)	Median Inhalationtime (sec)	Median Exhalationtime (sec)	puffs	Single puff	Short cluster	Medium cluster	Long cluster
HTP 1	02,200	02,133	01,800	15	2	3	1	0
HTP 2	01,667	01,475	02,146	39	0	0	0	3
HTP 3	02,053	02,421	01,474	19	0	4	1	0
HTP 4	01,625	01,000	01,000	32	2	7	1	0
HTP 5	01,535	01,105	01,098	43	0	0	0	3
HTP 6	01,829	02,329	01,284	70	0	0	0	5
HTP 7	01,429	01,557	01,029	70	0	0	0	4
HTP 8	01,987	01,382	03,079	75	0	1	2	4
HTP 9	02,875	01,325	01,088	80	0	1	0	5
HTP 10	01,386	01,025	01,012	83	1	1	1	5
HTP 11	01,088	01,088	01,000	34	6	4	1	1
HTP 12	01,952	01,345	01,631	84	1	2	3	4
HTP 13	01,320	01,000	01,000	25	4	2	2	0
HTP 14	02,327	03,538	01,731	52	0	2	2	2
HTP 15	02,532	02,274	01,935	62	0	2	4	2

Table S9. Puff topography in the CC group.

Participant	Median Mouth time (sec)	Median Inhalationtime (sec)	Median Exhalationtime (sec)	puffs	Single puff	Short cluster	Medium cluster	Long cluster
CC 1	02,145	03,691	01,911	55	0	0	2	3
CC 2	01,551	01,286	01,061	49	1	0	0	3
CC 3	01,824	01,939	02,353	34	3	10	1	0
CC 4	01,516	02,129	01,281	31	0	0	1	2
CC 5	02,350	02,075	01,200	40	0	4	1	2
CC 6	01,826	02,543	01,261	46	0	0	4	1
CC 7	01,385	02,897	01,538	39	10	10	0	0
CC 8	01,686	02,400	01,057	35	0	0	3	0
CC 9	01,789	02,605	01,368	38	0	0	4	0
CC 10	02,568	03,973	01,351	37	0	2	2	0
CC 11	01,667	01,771	01,250	48	0	0	0	4
CC 12	01,449	02,265	01,408	49	0	2	4	1
CC 13	01,176	01,157	01,000	51	1	1	0	3
CC 14	02,125	03,312	01,042	48	2	11	2	0
CC 15	01,743	01,914	01,200	35	0	4	2	0

5. Side effects

Cardiovascular side effects are displayed in Figure S1. Other inquired side effects were recorded at baseline, at 30 min and at 90 min, as shown in Figure S2.

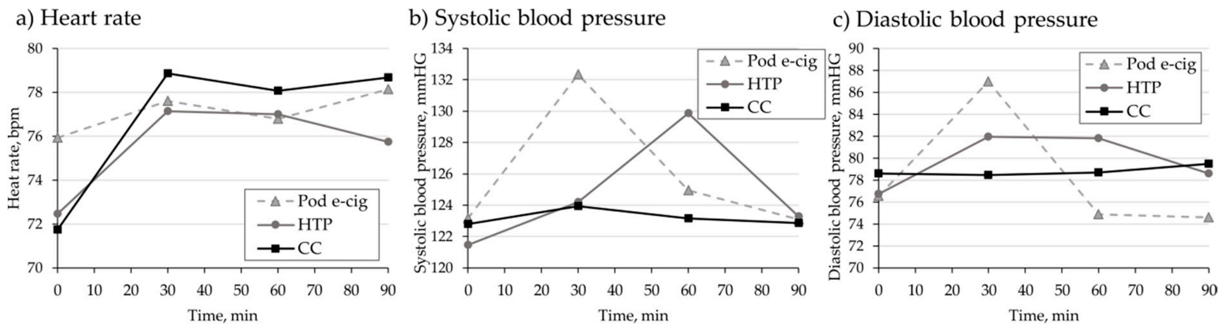

Figure S1. Changes in (**a**) heart rate, (**b**) systolic blood pressure, and (**c**) diastolic blood pressure in the three study groups: e-cigarette (pod e-cig) group, heated tobacco products (HTP) group, and conventional cigarette (CC) group. Results are presented as means.

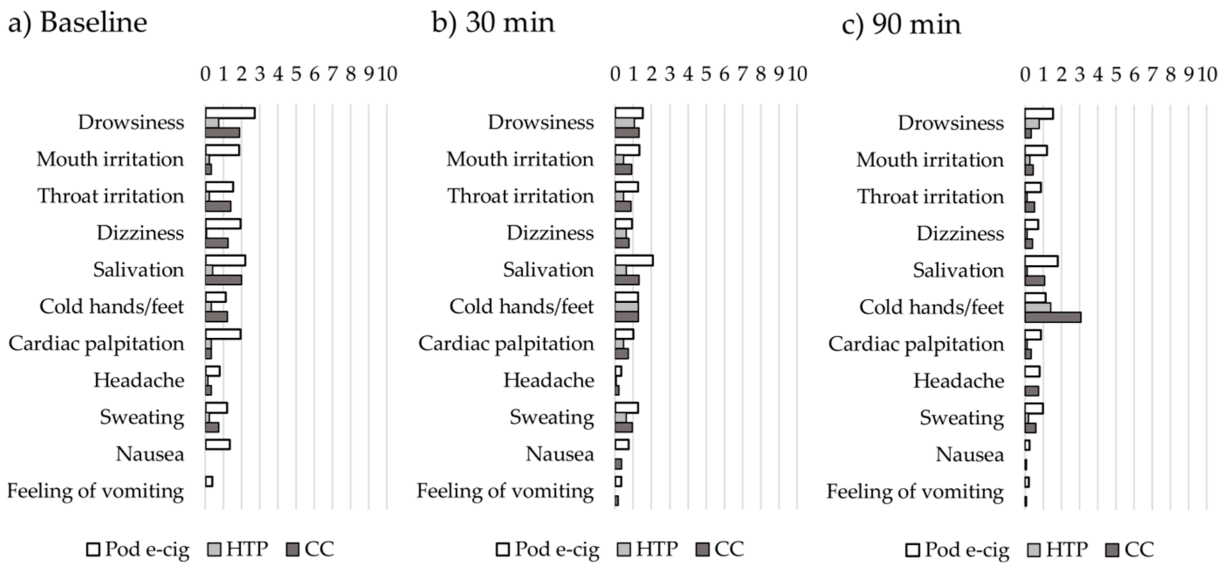

Figure S2. Side effects reported at (**a**) baseline, (**b**) 5 min or (**c**) 30 min on a visual analog scale (VAS) ranging from 0 (no effect) to 10 (strong effect) in the three study groups: e-cigarette (pod e-cig) group, heated tobacco products (HTP) group, and conventional cigarette (CC) group. Results are presented as means.
